# "Ejaculatory disorders and α_1_-adrenoceptor antagonists therapy: clinical and experimental researches"

**DOI:** 10.1186/1479-5876-4-31

**Published:** 2006-07-14

**Authors:** Marco Grasso, Flavio Fortuna, Caterina Lania, Salvatore Blanco

**Affiliations:** 1 Department of Urology Desio Hospital, Milan, Italy; 2 Department of Urology, San Raffaele Hospital, Milan, Italy

## Abstract

**Background:**

It is well known that the use of the α-adrenergic receptor antagonists in the BPH therapy may induce ejaculatory disorder. A review of clinical literature shows a greater incidence of ejaculatory disorder during the use of tamsulosin compared with alfuzosin. Anejaculation has been until now referred to retrograde ejaculation due to relaxation of prostatic and bladder neck smooth muscle tone. In a recent researches was evaluated the effect of tamsulosin and alfuzosin on rat vas deferent "in vitro", concluding that tamsulosin may "cause ejaculatory dysfunction by altering the progression and emission of sperm". An abnormal increase of contraction would be the cause of ejaculatory disorder. The aim of our paper is to compare human and rat vas deferens contractile activity and to evaluate with a clinical study if tamsulosin causes retrograde ejaculation disorder.

**Methods:**

We have revaluated the human and rat vas deferens contractile activity in vitro according to our experience and literature. We have also performed a clinical study on 10 patients (48–72 y) affected by anejaculation. Post-coital urine was examined to search spermatozoa.

**Results:**

Human and rat vas deferens activity is not comparable. Contractile activity induced by norepinephrin after tamsulosin incubation in rat prostatic vas deferens strips is similar to the contractile activity evoked by norepinephrin in human strips. Spermatozoa were found in post coital urine of 6 patients.

**Conclusion:**

In our opinion the treatment with tamsulosin may induce retrograde ejaculation but not other ejaculatory disorder due to abnormal sperm progression.

## Background

Alpha1-adrenoreceptor antagonists have been used for years in the treatment of lower urinary tract symptoms suggestive of benign prostatic hyperplasia (LUTS/BPH).

Over years, researches have identified different receptor subpopulations and consequently more and more selective alpha antagonist drugs have been developed, acting specifically on the lower urinary tract with lower effects on the cardiovascular system.

Currently available α_1_-Adreno Receptor (α_1_-AR) antagonists show an excellent efficacy profile in improving both the voiding (Qmax) and filling symptoms. The main difference among the α_1 _antagonists relates to the tolerability profile involving the cardiovascular (dizziness, arterial hypotension) and genital (anejaculation) systems [6].

As for ejaculatory disorders, anejaculation had always been thought to be caused by bladder neck relaxation. Recent *in vitro *researches [2,4] on rats' deferent ducts have suggested that the ejaculatory disorder would be secondary to anomalies in sperm progression due to the alteration in the contractile mechanism of the *vas deferens*.

We carried out two studies: one '*in vitro*' and one '*in vivo*', in order to verify this hypothesis.

## Methods

The *in vitro *study was carried out on fragments of human deferent duct taken from surgical portions (radical cystectomy, radical prostatectomy, orchiectomy).

The experimental model used is the following: a thermostatic bath, containing a segment of *vas deferens *fastened to the bath bottom, is connected by means of an extensible wire to the lever arm of an isometric transducer, generating an electric pulse to the microdynamometer recorder. The thermostatic bath maintains the temperature of the perfusion liquid at 37°C. The 'strip' of *vas deferens *is helicoidally cut so as to obtain a concentration being the sum of the longitudinal and circular muscle activity. The electric pulse transmitting the variations in isometric tension reaches the microdynamometer recorder: the sliding paper strip records the displacement of the pen induced by the change in the preparation tone. The abscissas axis indicates the sliding time corresponding to 6 mm/min., and ordinates axis shows the tension developed by the preparation, corresponding approximately to 1 cm/gram.

Fragments of *vas deferens*, taken from both epididymis and prostate, were stimulated with noradrenaline (dose-response curve) in order to evaluate the different mode of contractile response.

We also carried out a clinical trial on 10 patients (age range: 48–72 years) being treated with tamsulosin 0,4 mg for obstructive micturition disorders suggestive of BPH or bladder neck hypertonia and suffering from anejaculation. Patients reported normal erectile and ejaculatory activity, even if 7 out of 10 subjects had been noticing a clear reduction in the ejaculate volume for some years.

Patients were asked to urinate 15 minutes after ejaculation. Collected urines were centrifuged at 1500 RPM for 5 minutes. The sediment was evaluated by using a microscope (40×) in order to detect the presence of sperm cells.

## Results

**Figure 1:**
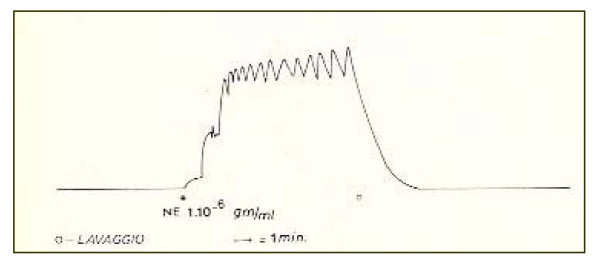
Response of an "epididymal" portion of the human vas deferens to a stimulation with noradrenaline (at the dose of 1.10–6 gm/ml). The tonic component prevails over the phasic one.

**Figure 2:**
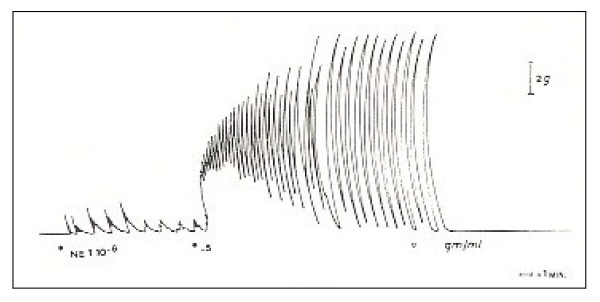
The typical response of the "prostatic" deferent duct has been characterized by an initial tonic-phasic "mixed" activity, immediately followed by a phasic activity marked by rapid strong "twitch-like" contractions. These latter may suggest the presence of a mechanism recruiting muscle cells, as if the vas deferens has a pace-maker action which is morphologically quite similar to the "twitch" that can be highlighted by electric stimulation.

The tonic component has prevailed over the phasic one in the response of the epididymal portion [Fig. 1]. On the contrary, the typical response of the prostatic deferent duct has been characterized by an initial tonic-phasic mixed activity, immediately followed by a phasic activity marked by rapid strong "twitch-like" contractions. These latter may suggest the presence of a mechanism recruiting muscle cells, as if the *vas deferens *has a pace-maker action which is morphologically quite similar to the "twitch" that can be highlighted by electric stimulation [Fig. 2].

**Figure 3:**
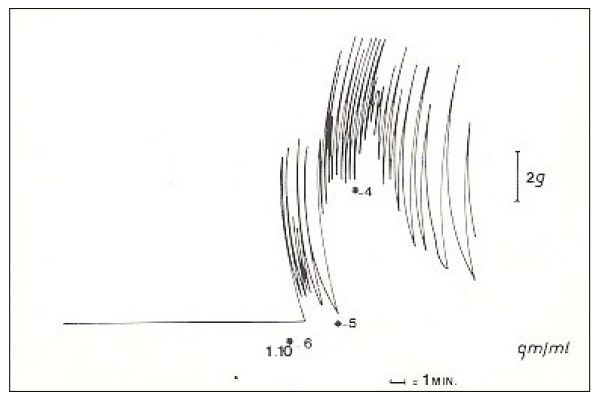
Following a dose-response stimulation with cumulative logarithmic doses of noradrenaline, it has been possible to observe that high doses of mediator markedly increase the basic tone without altering the maximum contraction (which is manifest in rapid phasic contractions), as if the "twitch" represents a contraction caused by the simultaneous excitation of all muscle cells and cannot be consequently exceeded.

Following a dose-response stimulation with cumulative logarithmic doses of noradrenaline, it has been possible to observe that high doses of mediator markedly increase the basic tone without altering the maximum contraction, just as if the "twitch" represents the contraction that is caused by the simultaneous excitation of all muscle cells and cannot be consequently exceeded [Fig. 3].

As for the clinical trial, sperm cells were detected in urines post-ejaculation in 6 out of 10 patients.

## Discussion

Alpha_1 _adrenoreceptor antagonists are commonly used in the treatment of LUTS/BPH. [4].

The presence of α-adrenergic receptors in the prostatic tissue was demonstrated more than 30 years ago by Caine [1]; over the latest years (1993), three α_1_-adrenergic receptor subtypes were discovered: α_1A_, α_1B_, and α_1D _[5,14].

α_1A _receptor subtype may be responsible of the contraction of the prostate smooth muscles; it is expressed in the prostate, trigone, bladder neck, seminal vesicles, deferent duct and distal ureteral tract with a clear prevalence over the other two subtypes. The α_1D _adrenoreceptor seems to be mainly present in the bladder and spinal cord.

On the basis of this different distribution, recent researches have suggested that α_1A _receptors mediate the voiding symptoms, whereas α_1D _receptors are involved in the development of the filling symptoms [13].

Several published clinical trials, have demonstrated the efficacy of the α_1_-AR antagonists in the treatment of LUTS. The meta-analysis – carried out by Djavan and Marberger [6] in 1999, was recently updated with studies retrived from a search in MEDLINE, BIOSIS and EMBASIS. This updated meta-analysis has unquestionably demonstrated the efficacy of the α_1_-AR antagonists in improving symptoms and Qmax, stressing again the key role of the alpha1 adrenoceptors in the development of LUTS [7].

Therefore, the impact of the α_1_-AR antagonists therapy on the elderly subjects' quality of life is quite evident, also in relation to the sexual function.

The ejaculatory function plays an important role in the sexual activity. Several authors have shown a high prevalence of ejaculatory disorders in men with LUTS [8]. Ejaculatory disorders have proven to be one of the most relevant side-effect, apart from the adverse events related to the cardiovascular system (dizziness, hypotension). Ejaculatory disorders have been reported more often by using the α_1 _antagonist tamsulosin [9].

In this regard, it seems particularly noteworthy to review data generated from large-scale observational studies.

The "Multinational Survey of the Aging Male" (MSAM-7) is a clinical trial carried out on 34,800 men (age: 50 to 80 years) in USA and in 6 European countries with the aim to define the correlations between LUTS and sexual problems, by using questionnaires and validated symptoms scales. For 12,815 men, results of the symptoms questionnaires were available.

### Results

- 83% of men were still sexually active

- 46% of these subjects had noticed a reduced ejaculate volume, while 5% reported the total absence of ejaculation

- The prevalence of ejaculatory disorders significantly increased with aging and severity of LUTS [10].

Another large-scale clinical trial (Asian Survey of aging males) was carried out in 5 Asian countries on 1,155 men aged between 50 and 80 years in order to establish the prevalence of LUTS and sexual disorders, as well as their correlation. The Authors observed that ejaculatory disorders (68%) were slightly higher than erectile ones (63%) and most men (52%) considered the ejaculatory dysfunction as the most bothersome problem [11].

Several hypotheses have been suggested about the correlation between LUTS and ejaculatory disorders; however, no definitive causal factor has been defined yet.

Some Authors have suggested that the increase in noradrenergic activity, observed in men with bladder emptying disorders, may interfere in the normal processes of erection and ejaculation [10].

Other authors have supposed that physical changes associated with BPH may mechanically alter the prostate and compress ejaculatory ducts, thus causing a reduced ejected volume and ejaculatory disorders [12].

Particularly noteworthy is a recent experimental work (Tambaro *et al*.), which has highlighted the different action of tamsulosin versus alfuzosin on the contractile activity of the deferent duct in rats, induced by noradrenaline (NE). Tamsulosin may induce an abnormal increase in the amplitude and frequency of contractions in the prostatic portion of the rat *vas deferens*, which may probably cause the ejaculatory dysfunction (anejaculation) due to unsuccessful progression and ejection of sperm [4].

In this study, 'strips' of the rat *vas deferens *were dissected at a distance of 1 cm from both prostatic and epididymal ends. Then, they were stimulated with increasing concentrations of NE until obtaining the dose-response curve with and without alfuzosin and tamsulosin.

NE has induced an initial rapid phasic contraction, followed by a slow tonic one with several intermittent 'spikes'.

Tamsulosin pre-treatment has induced an abnormal increase in intermittent spikes in both examined portions of the *vas deferens*. The greatest increase in contraction spikes has been observed in the prostatic tract (about + 550% in comparison with the control), whereas the tonic and phasic components of the contraction have been significantly reduced. Unlike tamsulosin, alfuzosin has not induced any significant increase in contraction spikes, even if it has shown a marked reduction in tonic and phasic components of the contraction. Therefore, it seems the response pattern is unaltered both in the epididymal and prostatic tracts of the rat *vas deferens*.

A possible hypothesis explaining this phenomenon at the prostatic level is the modulatory action of tamsulosin on Ca++ channels and the release of ATP; on the other hand, the inhibition of the response in the epididymal portion, induced by the nerve stimulation, may be due to the greater presence of α_1D _receptors, for which the affinity of tamsulosin is definitely higher than the one of alfuzosin.

On the contrary, our *in vitro *study has highlighted a key point, as also confirmed by other papers in literature [15]:

- the human deferent duct has a different contractile activity from that one of rats, as shown by Tambaro *et al*. In particular, the prostatic portion of the deferent duct has shown to respond to the stimulation with NE, with an intense phasic activity similar to that one described as 'abnormal' after incubation with NE in the rat *vas deferens*.

This difference in terms of response can be interpreted in different ways, considering the frequent inter-species variables.

Furthermore, the need of using 1 cm. of isolated organ in the experimental model to study the '*in vitro*' contractile activity has induced to evaluate the contractile response of a portion of the deferent duct which corresponds to the distal tenth of the human *vas deferens*. This latter also includes a large portion of the intermediate tract in a rat weighing 350 gr.

In that case, the contractile response cannot be considered representative of the juxtaprostatic tract.

## Conclusion

Based on the above-described data – on the evaluation of the different (NE-induced) contractile response in the human *vas deferens*, versus that one of rats, as well as the presence of sperm cells in postcoital urine in 6 out of 10 patients – we think it is reasonable to conclude that tamsulosin-induced anejaculation is caused by a bladder neck relaxation.

In fact, the low incidence of this phenomenon [6,7] is well-justified by anatomical changes in the prostatic urethra induced by BPH, whereas a direct effect on α_1_-adrenoreceptors on the vas deferens may be more common and reproducible in most patients treated with tamsulosin.

Furthermore, it would be necessary to make a final observation: ejaculatory disorders are related to an aged population suffering from LUTS/BPH – a condition that is associated with a high proportion of sexual disorders, as already mentioned.
